# Relationship between smoking, narcissism, and impulsiveness among young women

**DOI:** 10.1186/s40359-022-00809-5

**Published:** 2022-05-20

**Authors:** Semion Kertzman, Alex Kagan, Michael Vainder, Rina Lapidus, Abraham Weizman

**Affiliations:** 1 Department of Psychiatry, Adelson School of Medicine, Ariel University, Ariel, Israel; 2 School of Behavioral Sciences, Peres Academic Center, Rehovot, Israel; 3 The Program for Hermeneutics and Cultural Studies, Interdisciplinary Studies Unit, Bar-Ilan University, Ramat Gan, Israel; 4 Environics Analytics, Toronto, Canada; 5 Comparative Literature Department, Bar-Ilan University, Ramat Gan, Israel; 6 Sackler Faculty of Medicine, Tel Aviv University, Tel Aviv, Israel; 7 Research Unit, Geha Mental Health Center and Felsenstein Medical Research Center, Petach Tikvah, Israel

**Keywords:** Cigarette smoking, Narcissistic personality inventory (NPI), Barratt Impulsivity Scale (BIS-11)

## Abstract

Although the connection between smoking and individual differences has been recently recognized, the relationship between narcissistic personality traits and cigarette smoking has received less attention. The notion that personality traits can be associated with addictive behavior is influential in clinical practice. However, questions remain about specific interactions between smoking and personality characteristics that need empirical support to substantiate this hypothesis. This study thus identifies narcissistic and impulsive personality traits as precursors of smoking in a sample of tattooed individuals. In a cross-sectional study (N = 120), personality traits were assessed in young women (aged 18–35 years) using the narcissistic personality inventory and the Barratt Impulsivity Scale (BIS-11). The current study, using the regression analysis, has clearly demonstrated that young women who smoke have different personality characteristics as compared with women who do not smoke.

**Supplementary Information:**

The online version contains supplementary material available at 10.1186/s40359-022-00809-5.

## Introduction

Cigarette smoking is a major public health problem, and is the main cause of morbidity and mortality in industrialized countries [1]. There are over a billion smokers worldwide and among persistent cigarette smokers about 50% will eventually be killed by tobacco-related diseases [2]. But if smoking is an unhealthy behavior, why do people smoke?

Young adulthood is a critical risk period for the initiation of tobacco use [3]. Despite global trends indicating an overall decline, cigarette smoking is increasing among women of reproductive age in high-income countries [1]. The increased rate of smoking in adolescents coincides with the proposed trend of an increased sense of grandiosity among adolescents in the twenty first century [4, 5]. This trend has been coined “Generation Me” by observers [6].

Narcissism has been conceived as a personality [7]. Such a dispositional trait involves a sense of entitlement of privileged status over others, the belief that one is unique and more important than others and an excessive need for approval and admiration from others to feed the grandiose—but ultimately vulnerable—self [8]. People can engage in potentially health-damaging behavior such as smoking if they believe this behavior will make them appear sexy and cool [9]. Along these lines, it was found that in both males and females (sample of 72,435 students), subjective body image had a greater effect on daily smoking than body mass index [10]. Narcissists tend to enhance their self-image by purchasing products, especially symbolic, exclusive, and personalized ones [11, 12]. Others have suggested that specific aspects of narcissism rather than an increase in overall narcissism levels have shifted over generations. Thus, it is crucial to examine narcissism as a multi-faceted construct [13].

One personality characteristic that is robustly associated with smoking is impulsivity [14–16]. Narcissism, similar to smoking, is also associated with impulsivity [17]. A meta-analysis of 23 studies on the correlation between impulsivity and narcissism generated a mean value of r = 0.41. Researchers suggested that impulsivity is involved in narcissists’ risk-taking behavior [18]. It was demonstrated previously that a high impulsivity level is a key factor in "addictive personality" [19]. Research on current smokers has focused on psychological risk factors for both smoking initiation and nicotine dependence. Individual differences such as higher neuroticism, higher extraversion, lower conscientiousness and low agreeableness were associated with increased probability of cigarette smoking [20]. Certain personality traits such as low self-control, high resistance to rules, and sensation seeking can influence many smokers to persist in their use and have caused great difficulty in quitting [21–23]. Smokers are more likely to engage in high risk behaviors. Studies have found that smokers associated with traffic accidents [24], unsafe sexual behavior [25] are less likely to wear seatbelts [26], more prone to substance and alcohol abuse [27], and gambling addiction [28] than non-smokers. Overlap with a wide range of these behaviors can be related to other common factors such as impulsivity and narcissism that can be associated with a wide range of externalizing behaviors, although impulsivity and narcissism may reinforce each other as they may represent different contexts in smoking behavior. To our knowledge, the role of narcissism in smoking behavior has received little attention in the literature. The association between narcissism, impulsivity, and tobacco smoking has not been studied previously. It may be possible that both narcissism and impulsiveness contribute independently to smoking, and only impulsivity explains the relationship between narcissism and smoking.

This study explores the cross-sectional relationships between narcissistic personality characteristics and impulsiveness. These traits of young women and smoking behavior will be measured using the narcissistic personality inventory (NPI) [17], and the Barratt Impulsivity Scale (BIS-11) [29].

### Study hypothesis

We used a current study to test the hypothesis that smoking is associated with other factors that contribute to externalizing behavior. In this study, we compared the BIS-11 and the NPI of women who smoke and those who do not.

We predicted that: (1) Women who smoke would have higher total score on the NPI than women who do not smoke (2) Female smokers and non-smokers would show different profiles of narcissism facets; (3) Women who smoke would report higher impulsiveness than women who do not, as measured by the BIS-11; (4) There would be a significant correlation between the NPI and the BIS-11 scores among smokers but not in female non-smokers; and (5) There is difference between the relative contribution of narcissism and impulsiveness to smoking behavior. To the best of our knowledge this is the first empirical study on the conceptual links between narcissism and smoking behavior. In addition, the study evaluates the interaction between narcissism and impulsiveness in female smokers.

## Method

### Participants

The participants in the current study were included in our previous study on decision making processes in tattooed and non- tattooed women [30] entailed recruitment through a variety of arenas—social networks (Facebook), notices at the Bar Ilan University, and word of mouth. The study was approved by the review board at Bar Ilan University (Ramat Gan, Israel). NPI and BIS-11 scales [31] were used to analyze women only, thus avoiding gender differences.

We estimated sample size based on Total BIS 11 score. From the literature review we know that the standard deviation of Total BIS 11 score is in a range of 9.5–10.5. Using 95% confidence level and the margin of error equal 10% we estimated, using sample size formula, that the sample of 39 smoking women is needed to answer our study questions. We also wanted to reflect Israel Health Ministry information about women smoking rate in Israel. The smoking rate among women (age > 21) in Israel is 12.6%. Based on this information we decided to increase control group at least two times compared to the target group and stay with sample size of 39 for the smoking group and 81 for the control group.

Participants [39 smokers (age M-27.4, SD = 5.59)] and control [81 non-smokers (age M = 28.9, SD = 5.68)] groups were all from the Tel Aviv area, with similar socioeconomic backgrounds and were employed, or students (high school diploma or lower, BA, MA) or graduates.

Each participant attended an individual session (up to an hour and a half) that included a detailed explanation about the study, and its aims and then were asked to sign an informed consent.

All candidates underwent an interview to give researchers insight into their medical and psychiatric history, as well as their backgrounds and family life. Participants citing addictions (drug and alcohol abuse or dependence) other than cigarette smoking or psychiatric disorders (requiring treatment and/or medication), citing current or past DSM-IV-TR axis I psychiatric disorders were excluded from the study.

Over a five month period, participants volunteered and in lieu of payment were given a free psychological consultation and a professional assessment of their personality traits.

### Measures

#### Narcissistic personality inventory (NPI)

A measure that is recognized and used often to study personality psychology in narcissism is a 40 item measure (M = 17.85, SD = 7.6, α = 5.86) [17], known as NPI, which has been corroborated based on the use of a wide array of criteria [32]. Based on the DSM-related definition, Narcissism is defined as a continuum with extreme manifestations representing pathological narcissism, while the less extreme forms indicate nonclinical narcissistic personality traits [33]. NPI is a mix of both adaptive and maladaptive content connected to psychological resilience on one hand, and impaired personal relationships on the other [13].

All NPI scales were required as the total scores are a compilation of all the responses to the various items [34], encompassing various dimensions of personality. To this end, seven NPI scales—authority, exhibitionism, superiority, vanity, exploitativeness, entitlement and self-sufficiency [17]—were applied.

The reliability of the full scale internally is 0.83, with 7 subscale reliabilities of 0.50–0.73 [17]. The full scale after 13 weeks has high test–retest reliability (r = 0.81), with the test–retest on the subscales being lower (range: 0.57–0.80) [35]. In the Hebrew version of the questionnaire that was developed [36], analysis of the normative population demonstrated similar findings to the English questionnaire. Cronbach’s alpha was used to test the accuracy of the Hebrew version—0.9—and the validity scale was 0.88 [36].

#### Barratt impulsiveness scale leven edition (BIS-11)

This 30-item self-report measures impulsiveness yielding three subscales and a total score [37], is the most commonly used in both research and clinical settings [29]. The 30 statements are assessed by participants using a 4-point Likert scale that ranges from 1 to 4: 1 = never/rarely, 2 = sometimes, 3 = frequently and 4 = almost always/always.

The total scores range from 30 to 120. A higher total score indicates a higher self-reported level of impulsivity.

The 3 s order factors: Motor Impulsiveness (MI), reflect action without any forethought ("I do things without thinking"; 11 items; α = 5.52), Attentional Impulsiveness (AI), which reflects a reduced ability to maintain attention toward a stimulus ("I concentrate easily"; 8 items; α = 5.66), and Non-Planning Impulsiveness (N-PI), which reflects an emphasis on the present ("I plan tasks carefully"; 11 items; α = 5.66); [37]

#### Analysis

Data were analyzed using SAS 9.1 software for Windows. All analyses used two-tailed levels of significance. In the first stage, the univariate analysis was conducted to compare group differences in demographic and personality characteristics. In the second stage the multivariable logistic regression analysis was performed to assess the relationship between smoking and significant variables identified in the first stage.

The Shapiro–Wilk test was used to check whether or not variables of interest follow a normal distribution. Based on Shapiro–Wilk test outcome, a decision was made to use non-parametric Wilcoxon rank sum test to investigate differences in socio-demographic and personality characteristics measured on continues and ordinal scales.

As a measure of effect size for non-parametric statistical test the r-value, calculated as Wilcoxon test statistic divided by the square root of the number of observations, was used [38]. Multivariable logistic regression models were built using backward variable selection techniques, considering variables with a univariate *p* value ≤ 0.05 as potential independent risk factors. A p-value of less than 0.05 was considered statistically significant. The odds ratios (OR) with 95% confidence intervals (CI) were assessed for each predictor. A c-index was calculated to evaluate model discrimination, and the Hosmer–Lemeshow test was applied to evaluate model calibration.

## Results

A Shapiro–Wilk test was performed for demographic and performance metrics and showed that the distribution of these variables departed significantly from normality.

Wilcoxon rank sum test revealed that smoking women were significantly less educated than non-smoking women (Z = 3.56, *p* = 0.0004) with a measure of effect size r = 0.33, indicating a medium effect size. Age did not show significant differences between groups (Table 1).

**Table 1:** Between-group comparison of demographic characteristics of the study population: univariate analysis

Variable	Smoking (n = 39)		Non-Smoking (n = 81)		Statistics	
	Median (IQR)	Mean (SD)	Median (IQR)	Mean (SD)	Z	*p* Value
Education	12 (12–16)	13.8 (2.3)	16 (12–18)	15 (2.8)	3.56	0.0004**
Age	28 (22–33)	27.4 (5.6)	30 (26–33)	28.9 (5.7)	1.36	0.1716

Data are presented as median with interquartile range (IQR), mean with standard deviation (SD), Wilcoxon test Z statistic and p-value. *p < 0.05; **p < 0.01;

Univariate analysis showed significant differences between tobacco smoking women and non-smoking women in one NPI subscale Self-Sufficiency (Z = 2.41, *p* = 0.016) with a measure of effect size value r = 0.22, indicating moderate effect size. On average, the Self-Sufficiency scale in the group of women who smoke is higher than in the group of non-smoking women (Table 2). There was a significant difference in Total NPI score between two groups (Z = 1.98, *p* = 0.0486) of both groups were in the normal range with effect size value r = 0.18. Total NPI in the group of women who smoke is significantly higher than in the non-smoking group.

**Table 2:** Between-group comparison of the NPI scales: univariate analysis

Variable	Smoking		Non-smoking		Statistics	
	Median (IQR)	Mean (SD)	Median (IQR)	Mean (SD)	Z value	*p*
Vanity	1 (0–2)	1.26 (1.23)	1 (0–2)	1.31 (1.13)	0.31	0.7533
Exploitativeness	3 (2–4)	2.67 (1.28)	2 (2–3)	2.37 (1.16)	1.17	0.2404
Exhibitionism	3 (2–4)	2.85 (1.71)	2 (1–4)	2.36 (1.73)	1.44	0.1497
Superiority	3 (1–3)	2.39 (1.18)	2 (1–3)	2.03 (1.16)	1.61	0.1065
*Self*-*Sufficiency*	2 (2–3)	2.56 (1.25)	2 (1–3)	2.00 (1.32)	2.41	0.0160*
Authority	4 (2–6)	3.90 (2.22)	3 (2–5)	3.69 (2.14)	0.50	0.6185
Entitlement	2 (1–3)	2.51 (1.54)	2 (1–3)	2.01 (1.30)	1.55	0.1220
*NPI Total*	19 (13–22)	18.13 (6.41)	14 (11–20)	15.77 (6.07)	1.97	0.0486*

Data are presented as median with interquartile range (IQR), mean with standard deviation (SD), Wilcoxon test Z statistic and *p* value. *p < 0.05

A univariate analysis of BIS-11 scales revealed significant differences between women who smoke and those who do not in total (Z = 3.18, *p* = 0.0015) with a measure of effect size r = 0.29, indicating medium effect size and in BIS-11 subscale—motor impulsiveness (Z = 3.62, *p* = 0.0003) with r = 0.33, indicating also medium effect size. The group of female smokers had both these parameters at higher levels than the group of non-smoking women (Table 3).

**Table 3:** Between-group comparison of the BIS-11 scales: univariate analysis

Variable	Smoking		Non-Smoking		Statistics	
	Median (IQR)	Mean (SD)	Median (IQR)	Mean (SD)	Z	*p* Value
*MI*	21 (18–25)	21.31 (4.47)	18 (16–21)	18.19 (3.12)	3.62	0.0003**
AI	17 (13–19)	16.49 (4.04)	15 (13–17)	15.27 (3.24)	1.43	0.1521
NPLI	25 (21–28)	24.46 (3.93)	23 (20–26)	22.98 (4.08)	1.80	0.0726
*Total BIS-11*	60 (55–68)	62.26 (9.33)	55 (50–62)	56.43 (8.29)	3.18	0.0015**

Data are presented as median with interquartile range (IQR), mean with standard deviation (SD), Wilcoxon test Z statistic and *p* value. *p < 0.05; **p < 0.01

*BIS-11* Barratt Impulsiveness Scale, *MI* motor impulsiveness, *AI* attentional impulsiveness, and *NPLI* non-planning impulsiveness

The inter-correlations of the Self-Sufficiency and Motor impulsiveness personality predictors were not statistically significant. Pearson correlations were − 0.45 in female smokers and − 0.44 in non-smoker women.

The logistic regression analysis was conducted using Smoking as dependent variable and all significant variables identified by univariate analysis: Education, Self-Sufficiency, Total NPI score, Total BIS-11 and Motor impulsiveness as independent variables. The multivariable logistic regression demonstrated that education, Self Sufficiency and Motor impulsiveness were the variables that contributed significantly to a logistic regression model aiming to discriminate between patients that do or do not smoke (Table 4).

**Table 4:** Multivariate LR model on factors influencing outcome in smoking behavior

Variable	Coefficient	Wald Chi-Squared	*p* Value	Odds ratio	95% CI
Education	− 0.2797	9.1065	0.0025**	0.756	[0.630–0.907]
Self-sufficiency	0.3676	4.5736	0.0325*	1.444	[1.031–2.023]
Motor impulsiveness	0.2221	11.9563	0.0005**	1.249	[1.101–1.416]

*p < 0.05, **p < 0.01

Based on the results from the multivariable logistic analysis, an institutional model to predict tobacco smoking among young women was developed. The results showed that the probability p of the tobacco smoking is given by the equation:

$$\begin{aligned} \log \left( {{\text{p}}/1 - {\text{p}}} \right) & = - 1.8322 - 0.2797*{\text{Education}} + 0.3676*{\text{Self - Sufficiency}} \\ & \quad + 0.2221*{\text{Motor}}\;{\text{impulsiveness}}. \\ \end{aligned}$$

According to the model, a one-year increase in education can decrease the odds of smoking by 24%. The model also shows that increasing of the Self-Sufficiency and Motor impulsiveness scales by one unit raises the odds of being a current smoker by 44% and 25% respectively. The model had a c-statistic of 0.861, indicating moderately good discriminative ability. The Hosmer–Lemeshow Goodness of fit test was insignificant (*p* = 0.9757), suggesting that the model fit well.

## Discussion

This study found that increasing the Self-Sufficiency scale and Motor impulsiveness scale by one unit raises the odds of greater risk of smoking behavior. Yet, a one-year increase in education shows decreased odds of smoking behavior. Narcissism, as constructed by the NPI, contains a “confusing mix of adaptive and maladaptive content” [39].

Smoking in young women was associated with Self-Sufficiency—a dimension of personality embedded within the NPI that captures psychological resilience and social potency. Smoking is a visible act that leads to creation, affirmation, and reinforcement of social status. The repeated act of smoking is affirmed and reinforced. It is a signal to others AS an individual’s identification with the characteristics of the self as a smoker [40], the ability to act independently of norms, attitudes, peer behaviors, or beliefs about smoking [41] and differentiate self from non-smokers [42]. Thus, Self-Sufficiency can increase the likelihood of engagement in smoking behaviors as an aspirational aspect that is valued as independence and self-confidence. It may be possible that these results confirm the expectation that smoking in young women was used as a way to sense the self as being different, and to develop the identity that one’s social rank is higher, better, and privileged [43].

Impulsivity is a second mediator of smoking in young women. Between-group differences in the BIS-11 scores were smaller than in the Self-Sufficiency scale of the NPI. Table 1 shows that in women who smoke there was a significant higher score of Motor Impulsiveness scale than in women who do not smoke, in accordance with previous studies [44, 45]. These findings support the notion that the association between impulsivity and smoking is strong. Table 3 shows that for both females who smoke and those who do not smoke the Motor impulsivity scale of BIS-11 and Self-Sufficiency scale of the NPI relate to smoking behavior as separable personality characteristics that may have unique contributions to smoking behavior in young women.

The results of the present study replicate previous studies, showing that impulsivity was associated with smoking behavior using other measures such delay of gratification [46]. We also replicated previous findings that education was significantly associated with smoking [47]. Similarly, smoking in young women could confound the association between externalizing behavior problems and risky decisions [30]. Prior investigations show an association between getting tattoos and a higher rate of current tobacco smoking [30]. It is possible that smoking and tattooing are linked to other common factors such narcissism that can be associated with a wide range of externalizing behaviors. Smokers are more likely to be involved in risky behaviors such as traffic accidents [24], unsafe sexual behavior [25], and are less likely to wear seatbelts [26] than non-smokers. Relatedly, prior investigations found that persons with tattoos have a higher rate of alcohol and drug use [48], traffic accidents [49] and risky sexual behavior [50]. These findings suggest strong associations between smoking and tattooing. However, smoking was associated with the Self-Sufficiency dimension of NPI, while getting tattoos was related to the Exhibitionism measure of NPI [51].

## Limitations

Several limitations need to be considered when analyzing the results of our research. The study first of all focused on young women ages 18–35, narrowing the scope of our findings to one group, rather than different sample groups of females. Secondly, the “narcissist” term is relevant to those that have a (relatively) high NPI score for the narcissism trait, and was not used as a clinical label to examine comorbid personality disorders in the research participants. Thirdly, we did not analyze the number of cigarettes smoked by female smokers, precluding the idea that women highly addicted to smoking have larger elevations in their NPI and BIS-11 scores. Finally, we are unable to establish whether smoking causes personality traits, or personality traits cause smoking.

## Conclusion

The current study, using the regression analysis, has clearly demonstrated that Self-sufficiency was an important contributor to smoking. Impulsiveness was also an independent contributor to the smoking in young women. These findings support the notion that Self-sufficiency and Motor impulsivity are related to smoking. The relationship between narcissism and impulsivity warrants further investigation among young women who smoke, including the systematic assessment of the relationship between the number of cigarettes, coexistence with drug use, alcohol and behavioral addictions as well as the level of education. Overall, our results show that there is an association between certain personality characteristics and cigarette smoking. In trying to prevent tobacco use, it may well be of key importance to acknowledge the mutual influence between smoking on the one hand and self-sufficiency and impulsivity on the other.

## Supplementary Information

**Additional file 1.** 120 participants [39 smokers aged 18–35 (M-27.4, SD = 5.59)] and control [81 non-smokers age ranged 18–35 (M = 28.9, SD = 5.68). The Data includes results of Barratt Impulsiveness Scale-Eleven Edition (BIS-11) and narcissistic personality inventory (NPI).

## Data Availability

The data is available and attached to this manuscript.
